# Franz Nissl (1860-1919), noted neuropsychiatrist and neuropathologist, staining the neuron, but not limiting it

**DOI:** 10.1590/1980-57642018dn13-030014

**Published:** 2019

**Authors:** Marleide da Mota Gomes

**Affiliations:** 1 Federal University of Rio de Janeiro Institute of Neurology Rio de JaneiroRJ Brazil Associate Professor, Institute of Neurology, Federal University of Rio de Janeiro, Rio de Janeiro, RJ, Brazil.

**Keywords:** Franz Nissl, neuropathology, staining method, neuron theory, Franz Nissl, neuropatologia, método de coloração, teoria do neurônio

## Abstract

Franz Alexander Nissl carried out studies on mental and nervous disorders, as a clinician, but mainly as a pathologist, probably the most important of his time. He recognized changes in glial cells, blood elements, blood vessels and brain tissue in general, achieving this by using a special blue stain he himself developed - Nissl staining, while still a medical student. However, he did not accept the neuron theory supported by the new staining methods developed by Camillo Golgi and Santiago Ramón y Cajal. Nissl had worked with the *crème de la crème* of German neuropsychiatry, including Alois Alzheimer, besides Emil Kraepelin, Korbinian Brodmann and Walther Spielmeyer. He became (1904), Kraepelin's successor as Professor of Psychiatry and Director of the Psychiatric Clinic, in Heidelberg. Moreover, in 1918, the year before Nissl´s death, Kraepelin offered him a research position as head of the Histopathology Department of the newly founded “Deutsche Forschungsanstalt fur Psychiatrie” of the Max Planck Institute for Psychiatry, in Munich.

## RESUMO

Franz Alexander Nissl realizou estudos sobre transtornos mentais e nervosos, como clínico, mas principalmente como patologista, provavelmente o mais importante de seu tempo. Ele reconheceu mudanças nas células gliais, elementos sangüíneos, vasos sangüíneos e tecido cerebral em geral, realizando-o por meio de um corante azul especial desenvolvida por ele mesmo - coloração de Nissl, ainda como estudante de medicina. No entanto, ele não aceitou a teoria do neurônio esclarecida pelos novos métodos de coloração desenvolvidos por Camillo Golgi e Santiago Ramón y Cajal. Nissl havia trabalhado com o *crème de la crème* da neuropsiquiatria alemã, como Alois Alzheimer, além de Emil Kraepelin, Korbinian Brodmann e Walther Spielmeyer. Ele se tornou (1904), o sucessor de Kraepelin como professor de psiquiatria e diretor da Clínica Psiquiátrica, em Heidelberg. Além disso, em 1918, um ano antes da morte de Nissl, Kraepelin o convidou para um cargo de pesquisador, como chefe do departamento de histopatologia da recém-fundada “Deutsche Forschungsanstalt fur Psychiatrie” do Instituto Max Planck de Psiquiatria, em Munique.

**Figure 1:**
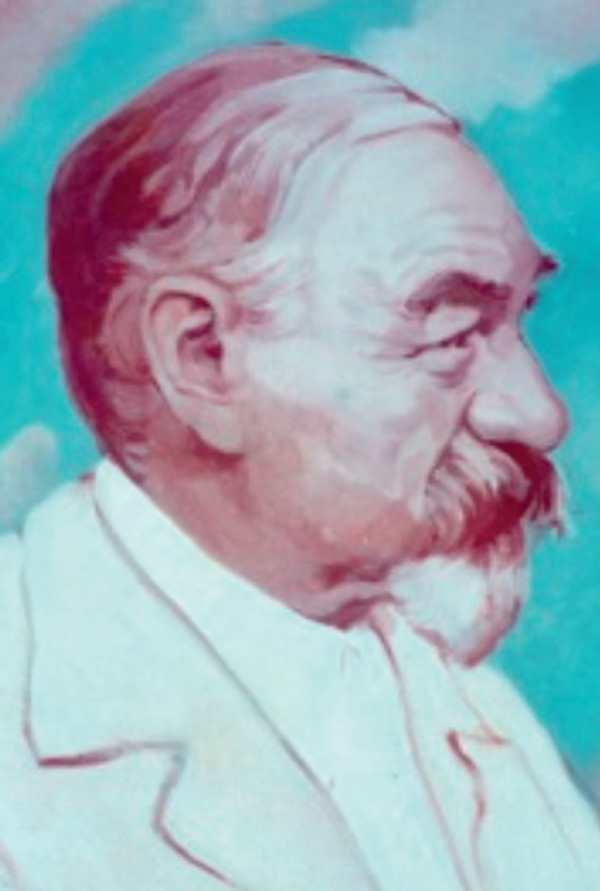
Franz Nissl (Frankenthal, 9 September 1860 - Munich, 11 August 1919) (Reproduced with the permission of the Neurological Museum - Institute of Neurology/Federal University of Rio de Janeiro, Brazil).

**Figure 2:**
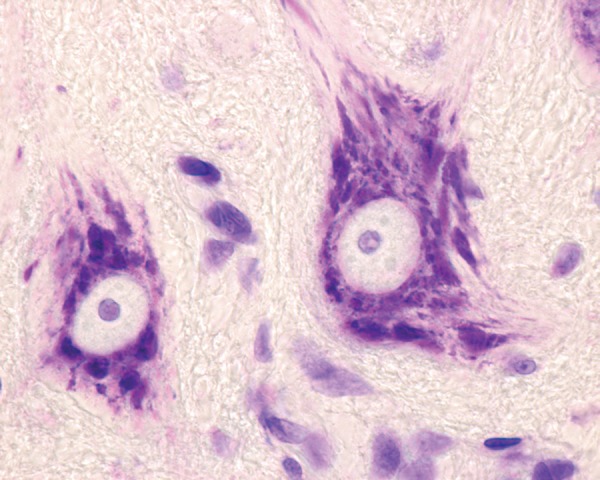
Nissl bodies in motor neurons located in the ventral horn of the spinal cord stained with cresyl.

In the second half of the 19th century and beginning of the next century, many research centers of neurology and psychiatry flourished in Germany, focusing on elucidating new disorders in these fields. Franz Alexander Nissl (1860-1919) (Figure 1) grew academically in this era and environment.^1^^-^5 He was a German psychiatrist, medical researcher, outstanding histopathologist/neuropathologist, while also a fine clinician, and become an illustrious representative of this era. He discovered a new technique for staining nerve cells (Figure 2), while still a medical student (1884), or more precisely a unique stain for characterizing neurons. The present paper provides an overview of his life and achievements, and mainly his most famous accomplishment, Nissl staining, as a way of honoring this forerunner of neuropathology.

## FRANZ NISSL, LIFE AND WORK

Nissl was surrounded by some of the most creative and effervescent minds of neuropsychiatry, in the best environment for this at the time, as it will be seen.

Nissl entered the Ludwig Maximilian University of Munich to study medicine. He later began training in psychiatry as an assistant to Professor Johann Bernhard Aloys von Gudden (1824-1886), a German neuroanatomist and psychiatrist who had students and assistants, such as Franz Nissl and Emil Kraepelin.^3^^,^^5^ While the former was a Catholic from South Germany and the latter, a Prussian raised in a Protestant family, Nissl and Kraepelin were close colleagues.^2^ The first, as a medical student (1884), was advised by Sigbert Josef Maria Ganser, Von Gudden´s assistant, to write an essay on the pathology of the cells of the cortex of the brain - later to become Nissl staining.^1^ Kraepelin was four years older than Nissl. Both researchers were hard workers, and the prize won by Nissl attracted more attention to Nissl from Kraepelin.^2^

Regarding Von Gudden, he served as consulting psychiatrist to Bavaria's royal family, but on June 13, 1886, King Ludwig and Gudden were both found dead in the water, maybe both assassinated.^1^^,^^3^ Because of Gudden´s position, he granted Nissl an assistantship at Furstenried castle, and he also continued with his neuropathological research at the Oberbayerische Kreisirrenanstalt Haar, in Munich, from 1885 until 1888.

In 1889, Nissl became a staff psychiatrist at the Städtische Irrenanstalt (Frankfurt City Asylum), under the new direction of Emil Sioli (1852-1922). A few months previously, he had also become acquainted with Alois Alzheimer.^1^^,^^5^ There, Nissl met neurologist Ludwig Edinger and neuropathologist Karl Weigert who was developing a neuroglial stain. Nissl did much of his pioneering work in collaboration with Weigert, besides Alois Alzheimer.^3^^,^^4^ This work stimulated Nissl to study mental and nervous diseases by linking them to observable changes in glial cells, blood elements, blood vessels and brain tissue in general. For the next seven years, Nissl and Alzheimer worked together conducting an extensive investigation into the pathology of the nervous system. In 1894, Alzheimer began to present his work on the histology of neurosyphilis, in close association with Nissl, and in 1898, Alzheimer revealed some uncommon variations in the brain tissue of a patient with senile dementia.^5^

In 1895, Emil Kraepelin, then head of the university psychiatry clinic in Heidelberg, invited Nissl to become an assistant physician of this facility. When Kraepelin left Heidelberg for Munich in 1903, Nissl became acting head of the clinic and was appointed, in 1904, Kraepelin's successor as Professor of Psychiatry and Director of the Psychiatric Clinic in Heidelberg. Under Nissl's aegis, the Heidelberg “Phenomenology School” flourished. As Aubrey Lewis later wrote of this period, apud Shorter:^5^ “[Nissl] was a conscientious clinician but he had little sympathy or understanding for the psychopathological approach to the problems of psychiatry. Nevertheless, he collected a group of able young people around him, who recognized the relative sterility of [the neuropathological] approach . . . and he gave them his puzzled approval to follow their lights”.^1^^,^^5^

As mentioned by Shorter,^5^ the concept of “nerve cell” comes from Nissl, and unlike Theodor Meynert in Vienna, Nissl understood that there were diverse types of nerve cells. Continuing his research publications on mental diseases, in 1904, Nissl embarked with Alzheimer on the first of a planned series on the histopathology of the cerebral cortex. This pioneering, seminal volume deals with the explanation of the pathological changes that take place in dementia paralytica.^5^ Thus, Nissl provided the classical account on the histopathology of this condition.^6^

In 1918, Kraepelin again invited Nissl to accept a research position at the Max Planck Institute for Psychiatry in Munich. Nissl, therefore, became head of the Histopathology Department of the newly founded (1917) German Psychiatric Research Institute (Deutsche Forschungsanstalt für Psychiatrie, or DFA)”. There, he performed research alongside Walther Spielmeyer and Korbinian Brodmann, whose cytoarchitecture was based largely on the use of the Nissl stain.^7^ Nissl was to lead the histopathology division until his death the following year. His colleagues of the DFA acknowledged that, according to Shorter, “Nissl is the founder and creator of the anatomy of mental illness”.^5^

As a clinician, Nissl popularized the use of spinal puncture, which had been introduced by Heinrich Quincke.^1^

Nissl was born in the small town of Frankenthal (Pfalz) in southwestern Germany, in the state of Rhineland-Palatinate, to Maria Haas and Theodor Nissl.^1^^,^^2^ His father taught Latin in a Catholic school and was preparing Nissl for priesthood, but he resisted and became a doctor. Regarding his mother, she had mental problems, so Nissl´s first contact with a mental asylum was linked to his own family. Nissl had a large, purple-pink birthmark on the left side of his face, maybe this was the reason why he was sometimes awkward in concealing the mark.^2^ In conclusion, he never married, and his life revolved entirely around his work. However, outside of his work, Nissl was a music devotee and a talented pianist. He is said to have had a keen sense of humor and enjoyed playing jokes. During World War I, he was assigned administrative duties at a large military hospital. The duties of teaching and administration, together with poor research facilities, meant Nissl had to leave many scientific projects unfinished. Overwhelmed with all these burdens, besides a severe kidney disease, Nissl died in 1919.

## FRANZ NISSL STAINING OF NEURON BODIES

Study of the anatomy of brain cells had to await the development over the years of a method to “fix” the tissue, without disturbing its structure, besides microtome to make very thin slices, in parallel with the development of the microscope. Thus, a fundamental further innovation in neurohistology, in the second half of the XIX century, was the introduction of stains that could selectively color some parts of brain tissue cells.^8^

As previously mentioned, Nissl developed a staining technique as a medical student, in 1884, and he “fixed the tissue in alcohol and stained with magenta red and followed it up with methylene blue and then, toluidine blue”, as reported by *Bhattacharyya.*^1^ This caused the nuclei of individual cells to stand out clearly, thus making their study possible in a manner that disclosed internal cell detail.^1^^,^^5^^,^^7^

This method allowed staining of important, hitherto unknown, structural neuron features, but constituents of nerve cells. The fragments which expressed dark blue were called Nissl substance and are now known as endoplasmic reticulum. Several eponyms bear his name, such as Nissl's methods, Nissl's stain, stains to show extranuclear RNA, and Nissl's substance. This last term is related to chromophilic substance in the form of granules found in the cell bodies and dendrites of neurons, but that is absent from axons. Regarding variations in these structures, Nissl bodies can show changes in various physiological conditions and, in abnormal situations, chromatolysis may occur.

Consequently, Nissl staining was extremely useful for distinguishing neurons and glia from one to another and for studying the arrangement, cytoarchitecture, of neurons in different parts of the brain.

The major disadvantage of using Nissl staining is that the detailed morphology of the cell is not stained. In 1873, the Italian neuroanatomist Camillo Golgi, used a silver chromate solution, now called Golgi stain, and was the first to distinguish that neurons had a cell body, axons and dendrites. Golgi recognized that neurons formed a continuous reticulum. However, Santiago Ramón y Cajal modified Golgi's method, and presumed that neurons were individual, discontinuous units that contacted other neurons through specific connections that were later called synapses. Cajal ´s idea that neurons are the functional unit of the nervous system came to be known as the Neuron doctrine.

Therefore, Nissl and his contemporaries fought for recognition of their respective views. Indeed, Nissl (1903)^9^ wrote a long and comprehensive summary of many differing opinions:^10^ “Die Neuronenlehre und ihre Anhänger: ein Beitrag zur Lösung des Problems der Beziehungen zwischen Nervenzelle, Faser und Grau” (“Neuron science and its followers: a contribution to solving the problem of nerve cell, fiber and gray relationships”). Indeed, Clarke & O'Malley (1996), apud Guillery,^10^ addressed the ‘awkward and long-winded' 1903 long paper on the Die Neuronenlehre (theory of neurons): “He (Nissl) described the postulated ‘nervösen Grau´ (neural grey) as a ‘specific neuronal, non-cellular component of the grey matter, which is firmly established even though we know nothing of its histological structure'”.

Thus, this coming of age of staining methods for the brain emerges in the neuronal theory, which draws on the silver staining of brain tissue developed by Golgi, besides the operating scheme and discontinuity of neurons by Cajal.

These achievements won the Nobel Prize for Golgi and Cajal (1906),^8^ but they remained rivals to the end.

**Figure 3:**
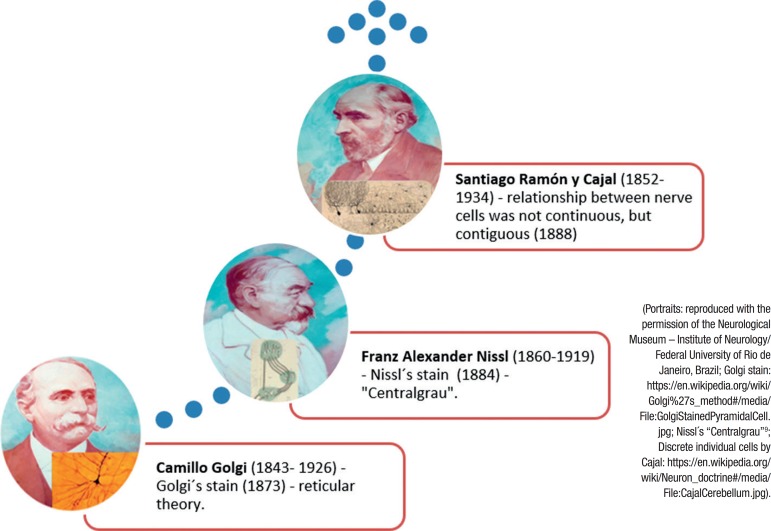
The succession of research into the structure of neurons defined modern study of the nervous system. Nissl refused to accept the nervous network of Golgi, but he presented the “Centralgrau” in the field of speculation-intercellular ‘grey’ between the axons and the dendrites - but not in the anatomical one. Cajal demonstrated the “neuron theory”.

In short, Nissl had the bad luck to be on the losing side of the controversy over the doctrine of the neuron, and he disproved neuronism in a clumsy way, and was also mistaken about neuron fibril function, as quoted by Chase^2^ (Figure 3).

Although Nissl's view disputing the neuron doctrine was not upheld, he is lauded as a pioneer of the anatomy of mental illness, at least of some neuropsychiatric diseases. Among his achievements, he reported for the first time in 1899, Rod microglia with elongated sausage-like soma that become oriented in certain directions in general paresis patients.^11^ He also developed his famous staining method, of extreme importance for studying the brain.
